# Precision and accuracy of four handheld blood lactate analyzers across low to high exercise intensities

**DOI:** 10.1007/s00421-024-05572-6

**Published:** 2024-08-19

**Authors:** Fredrik Mentzoni, Martin Skaugen, Ingrid Eythorsdottir, Stian Roterud, Espen Spro Johansen, Thomas Losnegard

**Affiliations:** 1 The Norwegian Olympic Sports Center, The Norwegian Olympic and Paralympic Committee and Confederation of Sports, Oslo, Norway; 2 Department of Physical Performance, Norwegian School of Sports Sciences, Oslo, Norway

**Keywords:** Endurance training, Endurance testing, Intensity zones, Threshold training, Measurement uncertainty, Reliability

## Abstract

**Purpose:**

To evaluate the precision and accuracy in measured blood lactate concentrations among four commonly used handheld lactate analyzers compared to two stationary analyzers.

**Methods:**

Venous blood samples were taken at exercise intensities ranging from low to high. The blood lactate concentration was measured simultaneously with four pairs of handheld lactate analyzers (two new units of each brand: Lactate Plus, Lactate Pro2, Lactate Scout 4, and TaiDoc TD-4289), and compared with two stationary analyzers (Biosen C-Line and YSI Sport 1500). Measurements were repeated for a range of blood lactate concentrations (measured with Biosen) from 0.88 to 4.89 mM with a median difference between measurements of 0.10 mM.

**Results:**

The mean relative differences to the Biosen analyzer were $-$7% (Plus), 7% (Pro), $-$10% (Scout), 42% (Tai), and $-$32% (Ysi). The residual standard errors after linear regression against Biosen were 0.18 mM (Plus), 0.20 mM (Pro), 0.22 mM (Scout), 0.15 mM (Tai), and 0.06 mM (Ysi). Accordingly, a blood lactate concentration of 3 mM measured with Biosen yielded 95% prediction intervals that were 0.72 mM (Plus), 0.80 mM (Pro), 0.87 mM (Scout), 0.60 mM (Tai), and 0.23 mM (Ysi) wide.

**Conclusion:**

Compared to our two stationary analyzers, the precision of the four handheld lactate analyzers evaluated in this study was poor. Among the four, Tai was the most precise; however, this analyzer had low accuracy with a substantial mean difference to the reference analyzer.

## Introduction

Blood lactate concentration can offer insights into an athlete’s exercise intensity as well as acute and long-term training status. However, the utility of blood lactate concentration depends on several factors (Swart and Jennings 2004). In addition to the considerable biological variation (Bagger et al. 2003), the significance of the measurement sample site (Raa et al. 2020), the specifics of the protocol employed (Bourdon et al. 2018), and the definitions applied (Jamnick et al. 2018), blood lactate assessments ultimately rely on the accuracy and precision of the lactate analyzers themselves. Earlier studies have suggested that the use of handheld analyzers is particularly challenging, as these devices are expected to have lower precision compared to their stationary counterparts (Swart and Jennings 2004; Bonaventura et al. 2015). Other studies suggest that the precision with handheld analyzers is generally good (Tanner et al. 2010; Crotty et al. 2021), and can be at least as good as stationary analyzers (Medbø et al. 2000).

Despite the potential challenges, it is common practice among endurance athletes to incorporate blood lactate measurements into their training and testing. The increase in popularity of the so-called “Norwegian method”, which relies on lactate measurements for intensity control in training (Casado et al. 2023; Tjelta 2019), underscores the relevance for lactate measurements in the field using handheld analyzers. Therefore, we evaluate four handheld analyzers currently used among athletes, comparing their accuracy and precision against two stationary analyzers that are commonly used in laboratories. Our goal is to assess the accuracy and precision of the handheld analyzers, and to highlight any differences between them. In the present study, we refer to accuracy as the ability of an analyzer to, on average, predict the true value as measured by the reference analyzer. Precision is referred to as the consistency of the analyzer in producing measurements that are close to each other, independent of any offset to the corresponding values on the reference analyzer. Ultimately, our assessment seeks to determine which of these handheld analyzers (if any) can be recommended for use, and to quantify the expected uncertainty associated with these devices, thereby offering guidance to practitioners relying on blood lactate measurements with handheld analyzers.

## Methods

### Lactate analyzers

This study evaluates the precision and accuracy of four pairs of handheld lactate analyzers: Lactate Plus (Nova Biomedical, USA), Lactate Pro2 (Arkray KDK, Japan), Lactate Scout 4 (SensLab GmbH, Germany), and TaiDoc TD-4289 (Taidoc Technology Corporation, Taiwan). The handheld analyzers are compared against two stationary reference analyzers: Biosen C-Line (EKF Diagnostic GmbH, Germany) and YSI Sport 1500 (Yellow Springs Instruments, USA). For simplicity, throughout the text, the analyzers are referred to as Plus, Pro, Scout, Tai, Biosen, and Ysi, respectively.

The considered analyzers differ in their methods of expressing blood lactate concentration. Lactate molecules in blood are found both in plasma and inside erythrocytes (red blood cells; RBC). All analyzers quantify blood lactate as a molar concentration, with units ${\text {mol}\cdot \hbox {m}^{-3}} = {\hbox {mmol} \cdot \hbox {L}^{-1}} = \hbox {mM}$ (millimolar). However, Biosen, Plus, Pro, and Scout hemolyze RBC and measure the total amount of lactate in plasma and RBC, expressing this relative to the volume of whole blood. In contrast, Ysi measures lactate only in plasma, expressing it relative to the volume of whole blood, whereas Tai measures only plasma lactate but expresses it relative to plasma volume. Importantly, at rest and during submaximal exercise, the lactate concentration in plasma is approximately 50% higher than in the erythrocytes (Foxdal et al. 1990). Consequently, compared to a given blood lactate concentration measured with Biosen, Ysi is expected to report a lower value, while Tai is expected to report a higher one. Due to its more common measurement technique, Biosen is used as the main reference in this study. Comparisons using Ysi as the reference are available in an external repository (Mentzoni 2024).

All lactate analyzers evaluated in this study measure the blood l-lactate concentration, [$\hbox {bLa}^-$], using an amperometric biosensor. The principle of this method is as follows: Utilizing an immobilized enzyme located either in the test strips (handheld analyzers) or between two membranes (stationary analyzers), lactate molecules in the blood sample are converted in an enzymatic reaction. All analyzers used in this study rely on lactate oxidase as the catalyzing enzyme, which oxidizes lactate to form pyruvate and hydrogen peroxide. Hydrogen peroxide is in turn oxidized, a process which releases electrons and creates an electrical current. The magnitude of this electrical current is directly proportional to the lactate concentration. Consequently, measuring the electrical current yields an estimate of the blood lactate concentration. More extensive reviews of this and other methods for measuring the blood lactate concentration are provided by others, cf. e.g., Nikolaus and Strehlitz (2008), Rathee et al. (2016).

The considered analyzers differ in blood sample size and measurement time. The volume of blood analyzed is 0.7, 0.3, 0.2, 0.8, 20, and 25${\mu \hbox {L}}$, while the measurement time is 13, 15, 10, 5, 20–45, and 30 s for Plus, Pro, Scout, Tai, Biosen, and Ysi, respectively.

The two stationary analyzers were calibrated according to their instructions. The Biosen analyzer was calibrated twice an hour against a low (3 mM) and a high (14 mM) solution. Additionally, the Biosen analyzer calibrated itself automatically every hour against a solution of 12 mM. The Ysi analyzer was calibrated against a 5 mM solution right before starting the measurement procedure. Additionally, a 15 mM solution was used to validate the linearity of the analyzer.

### Experimental design

A well-trained 35 year old male cyclist rode on a stationary trainer at four intensities corresponding to ratings of perceived exertion (RPE on the CR10 scale) of 1/10, 3/10, 5/10, and 6/10. For each intensity, after cycling at the set intensity for approximately 5 min, a venous blood sample was collected using a canula placed on the inside of the elbow. The sample was drawn into a vacutainer tube coated with lithium heparin. The mean power outputs in the last minute before taking the blood samples were 142 W (2.0 W $\hbox {kg}^{-1}$), 224 W (3.2 W $\hbox {kg}^{-1}$), 281 W (4.0W $\hbox {kg}^{-1}$), and 307 W (4.4 W $\hbox {kg}^{-1}$). The corresponding heart rate means were 120 bpm (63% of maximum heart rate), 149 bpm (79%), 156 bpm (83%), and 163 bpm (86%). These intensities were chosen as they cover a range of exercise intensities where measurements of blood lactate concentration are relevant (Casado et al. 2023).

Within 10 min of taking the blood sample, which was kept at room temperature ($\approx {22}\,^{\circ }{\hbox {C}}$) the whole time, the first measurement series of that intensity was conducted. A measurement series started by measuring the blood lactate concentration with the two stationary analyzers (Ysi first, then Biosen), referred to as pre-measurements. Blood from the sample was then measured “simultaneously” with four pairs of handheld lactate analyzers (in the order: Pro1, Plus1, Scout1, Tai1, Pro2, Plus2, Scout2, Tai2). Then, the blood lactate concentration was measured again with the two stationary analyzers (Ysi first, then Biosen), referred to as post-measurements. The mean of the pre- and post-measurements was used as reference.

For each of the four blood samples (i.e., intensities), 10 measurement series were conducted. The time between two consecutive measurement series was 4–10 min. The blood lactate concentrations (measured with Biosen) in the four series were 0.88–2.04 mM, 1.49–2.51 mM, 2.53–3.37 mM, and 3.85 to 4.89  mM. The rate of increase in blood lactate concentration with time was consistent with previous studies (Calatayud and TenÍas 2003; Zavorsky et al. 2021; Geyssant et al. 1985). Overall, this method yielded a range of blood lactate measurements from 0.88 to 4.89 mM with a median difference between consecutive measurements of 0.10 mM (measured with Biosen). A table containing all 480 measurements (four intensities, 10 measurement series, pre and post with the two stationary analyzers, and two units of each of the four handheld analyzers; $4 \times 10 \times 12$) is provided in an external repository (Mentzoni 2024).

To ensure consistency and an effective workflow, a single individual did all measurements with the handheld analyzers, and another did all measurements with the stationary analyzers. Both individuals are highly skilled with extensive experience in conducting lactate measurements. A video illustrating the procedure is provided in an external repository (Mentzoni 2024).

### Analysis

Python 3.11 was used for post-processing the measurements that were written into an Excel sheet during the measurement procedure. The pandas, numpy, statsmodels, scipy and pingouin packages were used for numerical and statistical analyses of the measurements, whereas matplotlib and seaborn were used for generating figures. The raw measurements and python scripts are provided in an external repository (Mentzoni 2024).

Residuals are calculated as the difference between the actual measured values, $y_i$, and the predicted values, $\hat{y}_i$, using linear least-squares regression for two sets of measurements (e.g., Plus vs. Biosen). The residual standard error (RSE) is the square root of the mean squared error (MSE) which is the sum of the residuals squared divided by the degrees of freedom,

1 $$\begin{aligned} \textrm{RSE} = \sqrt{\textrm{MSE}} = \sqrt{\frac{\sum _{i=1}^{n} (y_i - \hat{y}_i)^2}{n - 2}}, \end{aligned}$$

with *n* being the number of measurements.

Bland–Altman comparisons are performed for the two units within each handheld analyzer brand to assess the within-brand agreement. The within-brand results are presented in a table in the results section; within-brand Bland–Altman plots, as well as Bland–Altman plots for each analyzer brand against the Biosen analyzer, are made available in an external repository (Mentzoni 2024). The upper and lower limits of agreement (LoA) are calculated based on the *t*-score,

2 $$\begin{aligned} \text {LoA} = \overline{d} \pm t_{\alpha /2, \text {dof}} \times \textrm{SD}, \end{aligned}$$

here $\overline{d}$ is the mean of the differences between the two units and SD is the standard deviation of the differences. Setting the confidence level to ${95}\%$ and number of degrees of freedom to 39 ($n-1$ with $n=40$ measurements on each analyzer unit) yield

3 $$\begin{aligned} \text {LoA} = \overline{d} \pm t_{0.025, \text {39}} \times \textrm{SD} = \overline{d} \pm 2.02 \ \textrm{SD}, \end{aligned}$$

which is slightly larger than if using the *z*-score (1.96 SD).

## Results

**Fig. 1:**
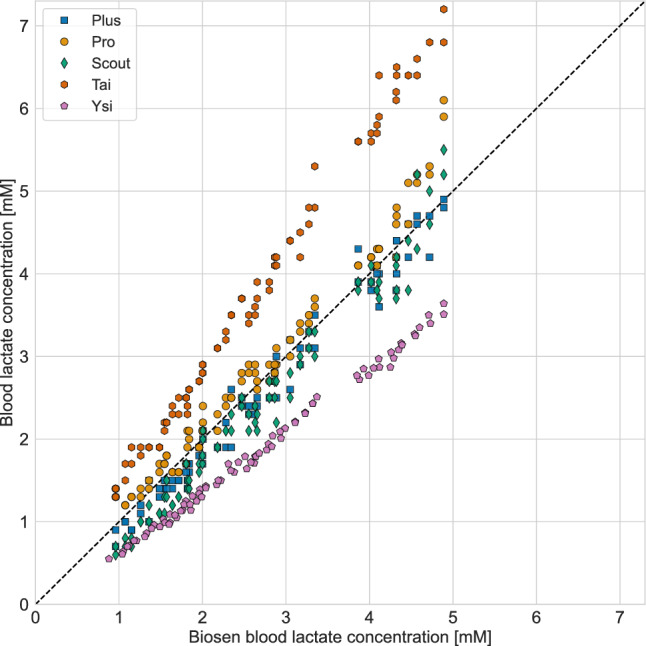
Blood lactate measurements. For the handheld analyzers, Plus, Pro, Scout and Tai, the *x*-value is the mean of the pre- and post-measurement with Biosen. For each *x*-value, there are two *y*-values per brand, one from each device. For Ysi vs. Biosen, both the pre- and post-measurements are plotted

In the following, we present the results of our evaluation of the accuracy and precision of the handheld analyzers using Biosen as the reference. Corresponding illustrations using Ysi as the reference are provided in an external repository (Mentzoni 2024).

An illustration of all measurements is presented in Fig. 1, in which the measured blood lactate concentration for each analyzer brand is presented as a function of the corresponding measurement with Biosen. The measurements include both analyzer units 1 and 2 plotted against the mean of the pre and post measurement with Biosen, totaling 80 measurements per analyzer brand. For Ysi vs. Biosen, both the pre and post measurements are plotted, in total 80 measurements. A table with all 480 measurements is provided in an external repository (Mentzoni 2024).

**Fig. 2:**
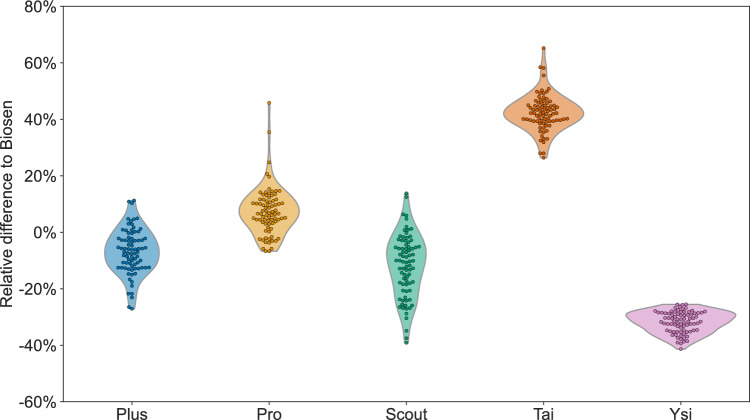
The measured blood lactate concentration with the four handheld lactate analyzers relative to the concentration measured with Biosen. Violin plots illustrate the distribution of measurements; markers illustrate each individual measurement

The measured blood lactate concentration relative to the measured blood lactate concentration with Biosen is presented in Fig. 2. The mean relative differences to Biosen were $-$7% (Plus), 7% (Pro), $-$10% (Scout), 42% (Tai), and $-$32% (Ysi). The 2.5- to 97.5-percentile ranges of measured relative differences were $-$23 to 10% (Plus), $-$6 to 25% (Pro), $-$35 to 6% (Scout), 28 to 58% (Tai), and $-$39 to $-$26% (Ysi).

**Fig. 3:**
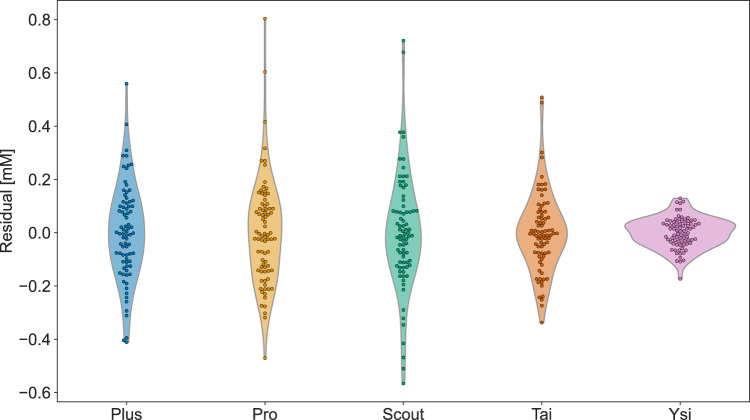
The residuals after linear regression against Biosen. Violin plots illustrate the distribution of residuals; markers illustrate each individual residual

The residuals, after ordinary least-squares linear regression against Biosen, are presented in Fig. 3. In addition to the handheld analyzers, the residuals after linear regression of the pre- and post-measurements of Ysi vs. Biosen are provided. The 2.5- to 97.5-percentile ranges of residuals were $-0.40$ to 0.31 mM (Plus), $-0.30$ to 0.42 mM (Pro), $-0.47$ to 0.38 mM (Scout), $-0.25$ to 0.31 mM (Tai), and $-0.11$ to 0.11 mM (Ysi).

**Fig. 4:**
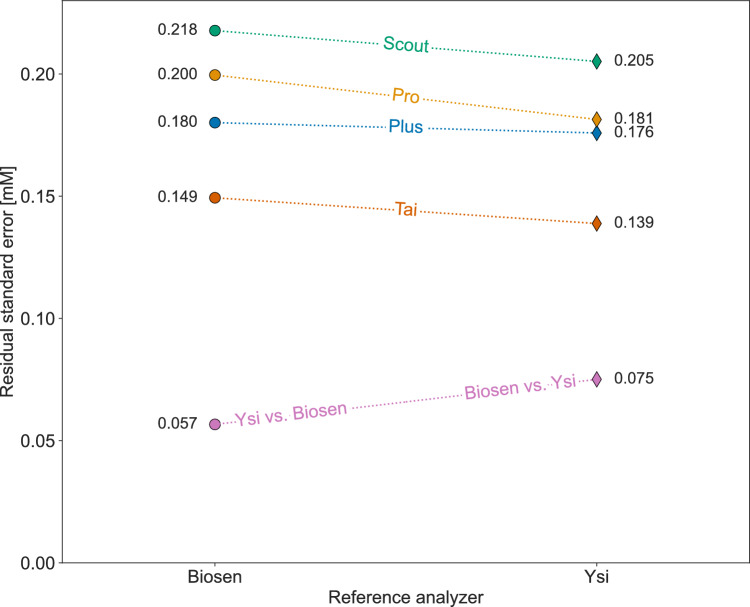
Residual standard error after linear regression against Biosen (left hand side) and Ysi (right hand side). The residual standard error was calculated based on the residuals of both units combined after linear regression of all measurements. Notably, for all four handheld analyzers, using Ysi as the reference yielded somewhat smaller residual standard error than if using Biosen

A presentation of the residual standard error for each analyzer using either Biosen or Ysi as the reference is given in Fig. 4. The residual standard errors were somewhat lower if using Ysi as the reference compared to using Biosen. A reason for the difference in residual standard error for Biosen vs. Ysi compared to Ysi vs. Biosen is due to the different scales in blood lactate concentrations, i.e., the measured blood lactate concentration with Ysi was on average 32% lower than with Biosen.

**Fig. 5:**
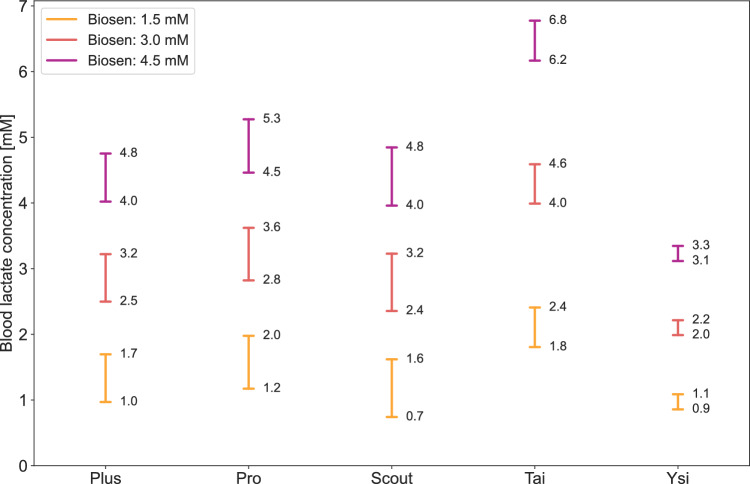
95% prediction intervals for three example blood lactate concentrations (1.5, 3.0 and 4.5 mM) measured with Biosen. The lower and upper limits of the prediction intervals are indicated, illustrating the expected range that a new measurement is likely to fall into with a probability of 95%

In Fig. 5, we illustrate the 95% prediction intervals for three example blood lactate concentrations (1.5, 3.0, and 4.5 mM) as measured with the Biosen analyzer. The lower and upper limits of these intervals are indicated, highlighting the range in which a new measurement is likely to be found with a 95% probability. Notably, with all analyzers considered in our study, the absolute range was relatively similar for low and high blood lactate concentrations within our range of measurements, meaning that the relative uncertainty was higher if measuring a blood lactate concentration at the lower end of our measurement range.

A summary of the within-brand comparison is presented in Table 1. The corresponding Bland–Altman plots are provided in an external repository (Mentzoni 2024).

**Table 1:** Bland-Altman comparison of unit 1 and unit 2 within each of the handheld analyzer brands

Analyzer	$\overline{d}$ (mM)	SD (mM)	Lower LoA (mM)	Upper LoA (mM)	LoA width (mM)
Plus	$-$0.09	0.26	$-$0.62	0.43	1.05
Pro	0.06	0.14	$-$0.23	0.34	0.57
Scout	0.06	0.26	$-$0.47	0.58	1.05
Tai	0.02	0.17	$-$0.33	0.37	0.69

The mean of the differences ($\overline{d}$), the standard deviation of the differences (SD), and the 95% limits of agreement (LoA) between the two units within the same brand are indicated. The limits of agreement are based on the *t*-score with 39 degrees of freedom, that is, the range, $\overline{d} \pm 2.02$ SD, is slightly larger than if using the *z*-score, i.e., $\overline{d} \pm 1.96$ SD

## Discussion

Our evaluation of the differences among four commonly used handheld lactate analyzers, and two stationary lactate analyzers, revealed considerable challenges with measuring blood lactate using handheld analyzers. The Plus, Pro, and Scout analyzers were reasonably accurate, with mean relative differences to Biosen of, respectively, $-$7%, 7%, and $-$10%. However, the precision of these analyzers was poor, with residual standard errors (RSE) of approximately 0.2mM. Accordingly, the 95% prediction intervals had relatively wide ranges of approximately $\pm {0.4}\,{\hbox {mM}}$. Tai showed slightly better precision (RSE of 0.15 mM, prediction intervals ranges $\pm {0.3}\,{\hbox {mM}}$), but the accuracy of this device was poor, requiring practitioners to consider a substantial mean relative difference to Biosen of 42%.

A likely reason for the large discrepancy in accuracy with Tai is the measurement technique applied, being the only handheld analyzer among the four that measures lactate only in plasma, not inside the erythrocytes. In general, low accuracy can be acceptable, particularly if the offset from the true value is known and can be adjusted for. However, compensating for an offset can be impractical, e.g., if one is used to refer to a specific exercise intensity corresponding to 3 mM, it might lead to misunderstandings and incorrect interpretations if this intensity corresponds to 5 mM with a different analyzer.

A limitation of our study is the use of our stationary analyzers as references, which are subject to measurement uncertainties and may not perfectly represent the true lactate concentrations. Switching the reference to Ysi from Biosen slightly reduced the residuals of the handheld analyzers, with an average reduction in RSE of 6%, cf. Fig. 4. This suggests that Ysi is a somewhat more precise reference analyzer than Biosen, introducing less uncertainty into the relation between the measured blood lactate concentration with the handheld analyzers and the reference analyzer. Nonetheless, this effect is relatively small compared to the uncertainty of the handheld analyzers, e.g., if using Ysi as the reference, the mean RSE of the four handheld analyzers is 2.3 times the RSE of Biosen vs. Ysi. The difference in RSE when comparing against Biosen or Ysi illustrates how the choice of reference can influence the perceived precision. Furthermore, any calibration offsets in the reference analyzer could lead to misinterpretations of the handheld analyzers’ accuracy. For example, if our main reference, Biosen, had been inaccurately calibrated with a constant offset of 0.2 mM, the mean relative differences for both Plus and Scout would be within ±3% of the reference lactate concentration, a considerably better result than what was found, cf. Fig. 2. An argument for using an analyzer like Biosen as the reference in our study is to provide practitioners with insights into how handheld analyzers compare against standard exercise laboratory analyzers. Notably, practitioners should be aware that using multiple analyzer brands can lead to complications, in particular due to considerable offsets in blood lactate concentrations from varying measurement techniques.

For each considered handheld lactate analyzer brand, we tested two units, making 40 measurements on each. The mean difference between the two units was less than 0.1 mM with all brands, indicating within-brand accuracy. However, the standard deviation of the differences was substantial. Consequently, the 95% limits of agreement between the two units cover a relatively wide range, which was more than 1 mM with two of the analyzer brands. This further confirms that the handheld analyzers have poor precision. We emphasize the limitations of our within-brand comparison, as we only tested two units of each brand.

Contrary to our findings, previous studies that have evaluated the precision and accuracy of handheld lactate analyzers have often concluded that these devices have good precision (Bonaventura et al. 2015; Tanner et al. 2010; Crotty et al. 2021; Medbø et al. 2000; Pyne et al. 2000; Hart et al. 2013). This discrepancy in conclusions can largely be attributed to what is considered “good enough” in terms of precision. Undoubtedly, the precision of the handheld analyzers in our study is poorer than that of our stationary analyzers. Note that despite the relatively large residual standard errors and correspondingly wide prediction intervals, the distribution of residuals is typically thickest around zero, cf. Fig. 3, suggesting that the most likely discrepancy (after correcting for an offset) is minimal. Nevertheless, the residual distributions of the handheld analyzers are relatively flat and wide, meaning that considerable deviations are also likely. In practical applications, if the goal is to determine if the blood lactate concentration is for instance 3 or 4 mM, all analyzers presently considered are likely applicable. However, for more nuanced measurements, like differentiating between 2.8 and 3.2 mM, the typical measurement uncertainty in the handheld analyzers can pose a problem. As argued by Jones et al. (2019), combining the measurement uncertainty of handheld lactate analyzers with the expected biological variation, the uncertainty associated with lactate measurements could make it challenging to determine whether an athlete is operating below or above a certain threshold intensity, such as the maximum lactate steady state. Consequently, if using blood lactate to evaluate training intensity, we recommend that it is used as part of a broader toolkit, rather than relying on it as the sole indicator.

## Conclusion

The precision of the handheld lactate analyzers evaluated in this study was considerably lower than that of our two stationary analyzers. Predicting a new measurement with 95% probability was associated with an uncertainty in precision of $\pm {0.4}\,{\hbox {mM}}$ with Plus, Pro, and Scout, and $\pm {0.3}\,{\hbox {mM}}$ with Tai. As such, Tai was the better of the four, however this analyzer had poor accuracy with a considerable offset to the reference analyzer, whereas the accuracy of the Plus, Pro and Scout analyzers was reasonably good.

## Supplementary information

Supplementary material is provided in an external repository on Figshare (Mentzoni 2024).

## Data Availability

All data are made available in an external repository on Figshare (Mentzoni 2024).
